# A Rare Case of Ascending Necrotizing Mediastinitis following Perforated Appendicitis: A Case Report

**DOI:** 10.70352/scrj.cr.25-0714

**Published:** 2026-02-26

**Authors:** Ju Hun Lee, Jong Eun Kim, Soon Ki Min

**Affiliations:** 1 Division of Vascular Surgery, Department of Surgery, Gachon University Gil Medical Center, Incheon, Korea; 2 Department of Radiology, Gachon University Gil Medical Center, Incheon, Korea; 3 Department of Trauma Surgery, Gachon University Gil Medical Center, Incheon, Korea

**Keywords:** necrotizing fasciitis, mediastinitis, appendicitis, retroperitoneal infection

## Abstract

**INTRODUCTION:**

Necrotizing fasciitis (NF) is a rapidly progressing soft-tissue infection associated with high mortality. While descending necrotizing mediastinitis (DNM) from cervical infections is well characterized, ascending necrotizing mediastinitis (ANM) originating from intra-abdominal sources is extremely rare.

**CASE PRESENTATION:**

A 66-year-old woman presented with right flank pain, fever, and leukocytosis. CT imaging revealed a pericecal abscess and retroperitoneal air associated with perforated appendicitis. Emergency right hemicolectomy with diverting ileostomy and extensive debridement were performed. Postoperatively, the patient developed empyema and mediastinitis due to retroperitoneal infectious spread through the diaphragm. A second-look operation confirmed progressive retroperitoneal necrosis without anastomotic leakage. Given the patient’s stability and prior surgical burden, mediastinitis was successfully managed with percutaneous catheter drainage (PCD). She made a full recovery following prolonged wound care and vacuum-assisted closure therapy.

**CONCLUSIONS:**

This case highlights an exceedingly rare form of ANM originating from appendicitis. In selected patients with adequate source control and clinical stability, minimally invasive management such as PCD may be an appropriate alternative to aggressive mediastinal surgery.

## Abbreviations

ANM
: ascending necrotizing mediastinitis
CRP
: C-reactive protein
DNM
: descending necrotizing mediastinitis
LRINEC
: Laboratory Risk Indicator for Necrotizing Fasciitis
NF
: necrotizing fasciitis
PCD
: percutaneous catheter drainage
VAC
: vacuum-assisted closure

## INTRODUCTION

Necrotizing fasciitis (NF) is a rapidly progressive disease characterized by aggressive infection spreading through soft tissue along the fascial planes, necessitating prompt and intensive treatment due to its high mortality rate. The incidence of NF has been reported to range from 0.3 to 15 cases per 100000 persons.^1,2)^ The mortality rate has been reported to range from 12% to 41.6%, underscoring its considerable fatality.^3,4)^ The risk factors for NF include advanced age (>60 years), diabetes mellitus, obesity, malnutrition, and immunocompromised states such as malignancy, steroid use, and organ transplantation.^3)^ NF can be classified by pathogen into 2 types: Type 1, a polymicrobial infection involving both aerobic and anaerobic bacteria, and Type 2, a monomicrobial infection, usually caused by Group A Streptococcus. NF can also be classified by infection site. The most commonly affected site is the limbs. If the perineum is involved, it is referred to as Fournier’s gangrene. NF may also involve the head and neck region, originating from odontogenic or pharyngeal infections. Additionally, cases have been documented in which NF originating from an intra-abdominal infection extends to the abdominal wall, chest wall, or even the inguinal region and thigh. If the infection progresses further and extends to the mediastinum and its surrounding structures, it is referred to as necrotizing mediastinitis. Particularly, when NF originates from the neck and spreads along the cervical fascia to the mediastinum, it is termed descending necrotizing mediastinitis (DNM) or descending NF. In contrast, an abdominal or retroperitoneal infection may ascend through the diaphragm, subsequently spreading to the mediastinum or chest wall. This condition, referred to as ascending necrotizing mediastinitis (ANM), has been rarely documented in the literature.

Diagnosis of NF requires a comprehensive assessment, incorporating clinical history, physical examination, and laboratory findings. A CT scan can also be utilized to aid in diagnosis. However, these modalities are insufficient for a definitive diagnosis, as surgical exploration remains the gold standard. These diagnostic limitations make early and accurate diagnosis particularly challenging. The cornerstone of treatment is urgent surgical debridement combined with the administration of broad-spectrum antibiotics. In severe cases, patients often require ICU management with resuscitation as needed.

We report a case of ascending colon perforation secondary to appendicitis, leading to infection extending into the retroperitoneal space and subsequently spreading along the diaphragm, ultimately resulting in empyema and mediastinitis. To the best of our knowledge, this is the first documented case.

## CASE PRESENTATION

**Fig. 1:**
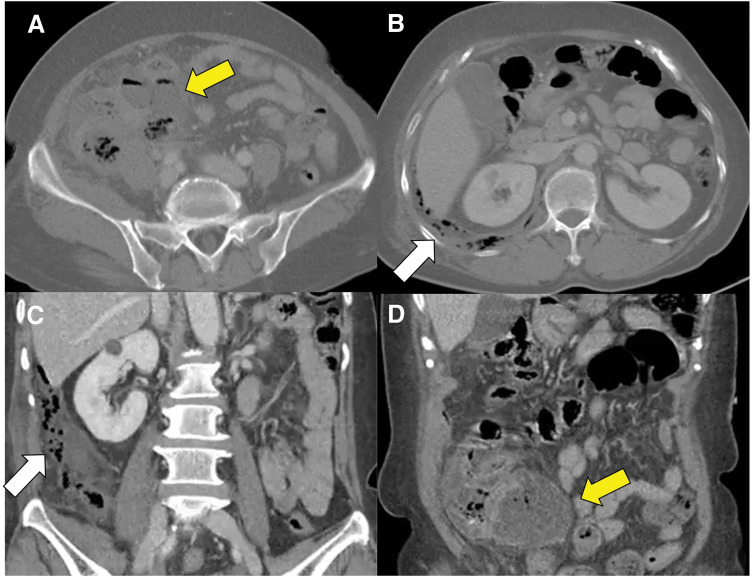
Enhanced CT images show a large 6.0-cm abscess (yellow arrows) in the pericecal area. Mottled air bubbles (white arrows) in the right paracolic gutter indicate pneumoperitoneum (**A**, **B**: axial; **C**, **D**: coronal).

The patient was a 66-year-old woman with no significant medical history. She was 145 cm in height and weighed 60.3 kg, with a BMI of 28.7 kg/m^2^ at the time of admission. She presented with right flank pain that began the day before admission, although she retrospectively reported a week-long history of mild dyspepsia and vague abdominal discomfort that she had initially overlooked. Upon admission, the patient exhibited leukocytosis (15.73 × 10³/μL), fever (38.7°C), and an elevated C-reactive protein (CRP) level (25.67 mg/dL). Abdominopelvic CT revealed a pericecal abscess secondary to cecal perforation (**Fig. 1**). Additional findings included wall thickening of the ascending colon and pneumoretroperitoneum, suggesting a widespread inflammatory process.

**Fig. 2:**
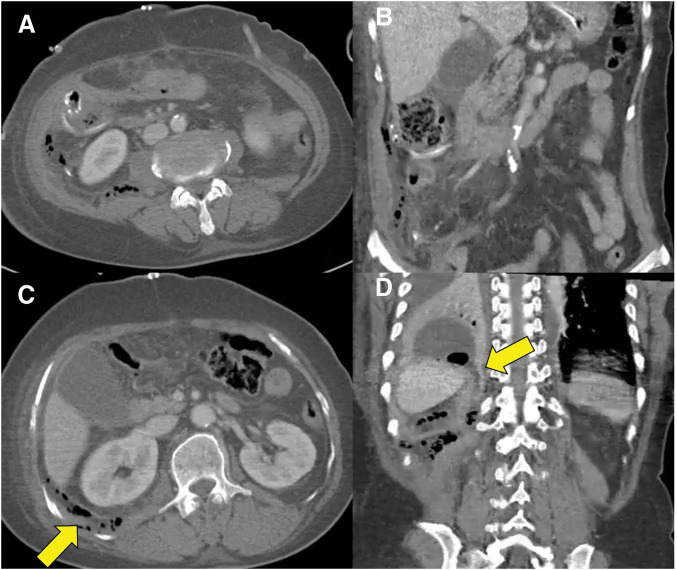
Postoperative CT shows small air-containing fluid near the hemicolectomy site (**A** and **B**). Empyema with suspected pleuro-retroperitoneal fistula is seen (yellow arrows) (**C** and **D**).

An emergency surgery was promptly performed, during which extensive phlegmon and necrotic tissue were identified around the cecum. While dissecting the right colon, perforation and adjacent necrotic changes were observed near the hepatic flexure of the ascending colon. However, intraoperative findings also revealed severe necrotic inflammation involving the appendix and cecum, with evident perforation in this area. Although the extent of inflammation made precise localization difficult, the pattern and anatomical distribution were suggestive of a primary perforation of appendicitis extending to the adjacent cecal wall. After performing thorough debridement and irrigation, a right hemicolectomy with formation of a diverting loop ileostomy was performed, and drains were placed in both paracolic gutters, pelvis, and perianastomosis site. Postoperatively, the patient was transferred to the ICU, where she received broad-spectrum antibiotic therapy with piperacillin-tazobactam and comprehensive critical care management. However, the patient continued to exhibit persistent leukocytosis, fever, and an elevated CRP level postoperatively, prompting a CT scan on POD 3. CT imaging revealed empyema, accompanied by mediastinitis (**Fig. 2**). Additionally, an air-containing fluid collection was observed near the anastomosis site, right posterior pararenal space, and subphrenic space. However, since the volume was minimal, and surgical drains had already been placed, percutaneous catheter drainage (PCD) was performed primarily for the empyema.

**Fig. 3:**
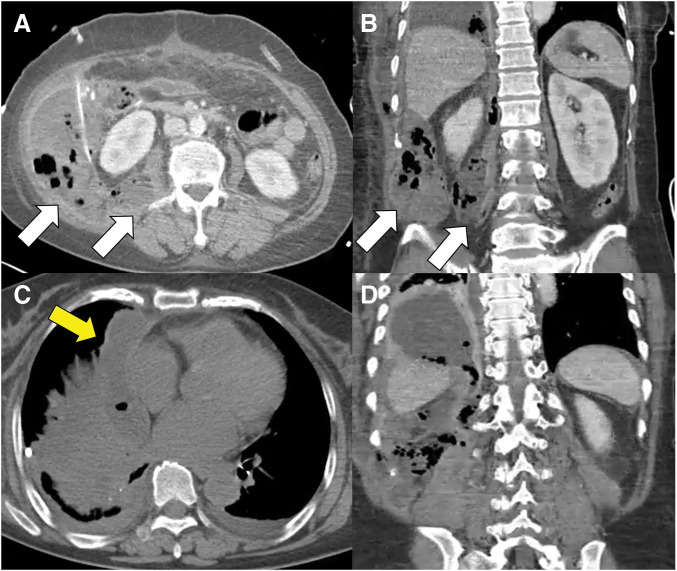
CT shows increased air-containing fluid around the hemicolectomy site (white arrows) (**A** and **B**). Worsening empyema persists despite drainage, with a newly visible paramediastinal gas-containing collection (yellow arrow) (**C** and **D**), suggesting mediastinitis.

**Fig. 4:**
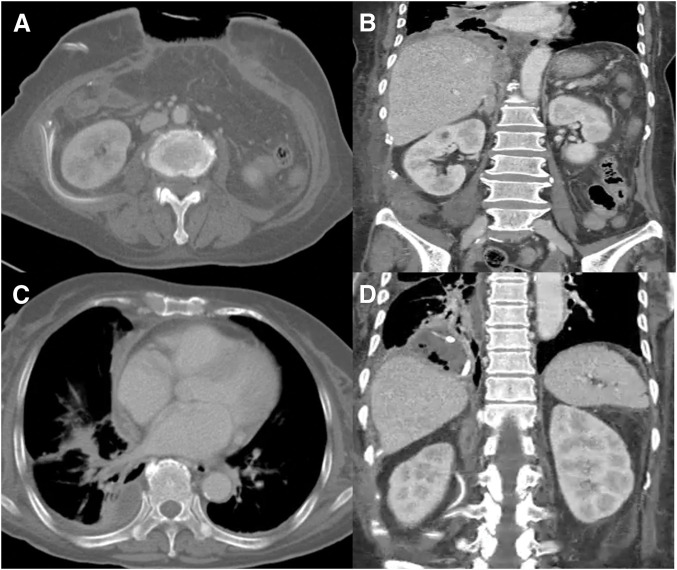
Follow-up CT images demonstrating interval improvement of the infectious process. Marked decrease in fluid collection at the operative site (**A**). Decrease in mediastinal and suprahepatic fluid collections (**B**). Reduction of pleural fluid with improvement of the empyema (**C**). Resolution of the pleural and mediastinal fluid collections with a patent PCD (**D**). PCD, percutaneous catheter drainage

Nevertheless, the patient’s condition did not improve, raising suspicion of an anastomotic leakage, which necessitated a second emergency surgery. Intraoperatively, the anastomosis site was found to be intact; however, necrosis had progressed to the retroperitoneal fat. The infection had extended to the adjacent abdominal wall, resulting in dissolution of the transverse abdominis muscle and hematoma formation. Accordingly, extensive debridement was performed to remove necrotic tissue from the abdominal wall, perirenal, pararenal, and subphrenic spaces, followed by thorough irrigation. Surgical drains were placed in key areas, and the surgery was successfully completed. A follow-up CT scan on POD 4 after the second emergency surgery revealed a fluid collection extending into the mediastinum, indicating further progression of the infection (**Fig. 3**). However, the patient’s condition was gradually improving, and lung PCD was functioning effectively. Considering the patient’s overall condition and the significant risks associated with repeated surgical interventions, we opted for close monitoring rather than additional surgery. Over time, the patient continued to recover, with a progressive reduction in drainage output from both the PCD and surgical drains. Follow-up CT findings correlated with these clinical improvements, confirming resolution of the infectious process (**Fig. 4**). Subsequently, meticulous wound management was performed, and a vacuum-assisted closure (VAC) system was applied to promote tissue granulation. The patient continued treatment in a general ward and was eventually discharged in a fully recovered state 3 months postoperatively. Six months later, she underwent a successful ileostomy reversal with no complications.

## DISCUSSION

### Pathophysiology and infection spread

Perforated retrocecal appendicitis often presents without typical peritoneal signs and makes early diagnosis challenging. As the condition progresses, the extent and severity of inflammation tend to increase and frequently lead to gangrenous changes. For this reason, the risk of developing uncommon but severe complications is increased. Under specific conditions, bacterial infections can spread along the fascia, resulting in NF.^5)^ Even cases of NF originating from an intra-abdominal infection and extending to the abdominal wall, chest wall, or thigh have been previously reported.^6,7)^ Our case represents a highly unusual presentation in which an intra-abdominal infection ascended via the retroperitoneal space, traversed the diaphragm, and ultimately extended into the mediastinum, a progression that has been rarely documented in the literature.

NF has been reported in cases as a complication of appendicitis, where infection extends to the abdominal wall,^5)^ as well as a complication of colon perforation, where the infection spreads to the thigh.^7,8)^ Similarly, cases have been documented following total abdominal hysterectomy, with NF involving the chest wall,^9)^ and after neck infections, where the infection progresses to the mediastinum.^10)^ These cases notably demonstrate a cranial-to-caudal progression and referred to as “descending” infections. In contrast, several cases, including ours, represent “ascending” infections. In these cases, intra-abdominal infections extend to the mediastinum, differing from the previously described ‘descending’ infections in terms of their directional spread.^11,12)^ A review of documented cases suggests that infection pathways can be categorized based on their direction of spread, broadly classified as ascending or descending.

Although retroperitoneal infection extending to the mediastinum is rare,^11,12)^ the present case represents the fifth documented instance of ANM originating from an abdominal focus.^12)^ Among the previously reported cases, outcomes ranged from early postoperative mortality^12)^ to successful recovery with surgical or minimally invasive management.^11,13,14)^ Especially, cases of NF originating from appendicitis have predominantly spread to the abdominal wall. To our knowledge, this is the first reported case in which appendicitis has directly led to ANM. Therefore, we present this case to share our experience in the surgical and interventional management employed during the treatment process.

### Treatment approach and surgical strategy

The decision to manage the mediastinal infection with PCD in our patient was justified based on multiple factors. The patient had already undergone multiple surgical interventions, significantly increasing the risks associated with additional operations. Therefore, although no established consensus exists on this approach, continuing with PCD instead of pursuing a high-risk surgical intervention was considered the appropriate course of action. This was supported by the patient’s favorable response to antibiotic therapy and effective drainage, which demonstrated clinical improvement.

The primary treatment for NF is urgent surgical debridement, accompanied by broad-spectrum antibiotic therapy as part of the standard management protocol. The fundamental principle of surgical debridement is the complete removal of necrotic tissue, ensuring thorough eradication of the infectious source. In our patient, all removable necrotic tissues in the abdominal wall and retroperitoneal space were thoroughly excised during the exploratory laparotomy. Preoperative CT imaging before re-operation did not clearly confirm the spread of infection to the mediastinum (**Fig. 2**), leading to the decision to limit debridement to the abdominal cavity. Over time, mediastinal infection was eventually identified. However, CT findings at that time suggested that the mediastinal infection was accompanied by inflammatory changes secondary to the adjacent empyema, reinforcing the appropriateness of PCD as a treatment approach (**Fig. 3**).^15,16)^

Cervical NF that originates from odontogenic or oropharyngeal infections in the neck and spreads along the cervical fascia to the mediastinum is referred to as DNM.^10)^ In such cases, rapid surgical drainage remains the primary treatment strategy, with PCD being considered as an additional option when feasible. Although there is limited discussion on the comparative efficacy of these approaches, Sumi et al. reported that the difference in mortality rates between surgical drainage and PCD is not significant.^10)^ In cases of DNM where the patient is assumed to be in a semi-Fowler position, gravitational force naturally drives the infection downward. However, in our case, the infection was spreading upward, and the primary source had already been eradicated with drainage well established. As a result, unlike in DNM, where the infection follows a natural downward drainage path and the drainage site is located downstream, this case presented a fundamentally different scenario. This key difference further supports the rationale for our chosen approach.

Considering the infection’s dissemination pathway, our case exhibits a strikingly similar pattern to previously documented instances of ANM.^11,12)^ From an anatomical perspective, infections spreading through the caval foramen are more likely to remain confined to the pleural space rather than disseminating further. In our patient, a comparable mechanism appears to have played a role in disease progression and treatment response. A previously documented case of ANM originating from emphysematous pyelonephritis was successfully managed using PCD alone.^11)^ Similarly, mediastinitis in our case was effectively controlled through drainage alone. Thus, this finding may further support the rationale behind our chosen treatment strategy.

### Infection pathway analysis

The infection pathway can be analyzed as follows: Initially, appendicitis led to abscess formation, which subsequently triggered widespread inflammation in the ascending colon. As the inflammation progressed, perforation of the ascending colon occurred, leading to the spread of infection into the retroperitoneal space. The infection ascended along the anterior fascia of the psoas muscle. Subsequently, it progressed from the psoas fascia to the diaphragm fascia, facilitating its upward spread through the caval foramen. Anteriorly, the fusion line between the fibrous pericardium and the respiratory diaphragm may have acted as a barrier, limiting further extension. Consequently, inflammatory exudate may have accumulated within the potential space between the pericardium and the parietal pleura, leading to fluid collection formation in the mediastinum. However, it is also possible that the mediastinitis developed as a secondary complication of empyema rather than as a result of direct infection spread through the aforementioned pathway.

On the other hand, the hypothesis that the infection originated from perforated appendicitis is supported by both histopathological and intraoperative findings. The pathology report confirmed acute gangrenous inflammation, abscess formation, and perforation involving the appendix and cecum. During surgery, severe inflammation and tissue necrosis were observed predominantly around the appendix and cecal region. No diverticula were identified in the segments of colon included in the resected specimen. In addition, no gross abnormalities suggestive of diverticulitis were noted in other segments of the colon during intraoperative inspection. Also, CT scans did not demonstrate any evidence of diverticular disease. Based on these collective findings, we determined that appendicitis was the most likely source of infection in this case. The apparent absence of gross necrosis in the intervening ascending colon can be explained by a patchy pattern of inflammatory spread rather than by a distinct pathology, which further supports appendicitis as the most plausible primary source.

In addition, pneumomediastinum originating from retroperitoneal infection has been reported, including cases secondary to acute appendicitis.^17)^ These reports emphasize that the retroperitoneal space and the mediastinum are anatomically connected compartments, allowing air and infection to spread between them. Such observations support the concept of a shared anatomical pathway through which intra-abdominal pathology may extend cranially. In our case, the infection appeared to follow a similar route, with progressive involvement along the retroperitoneal and fascial planes, reflecting advancement along this communicating pathway.

### Antibiotic therapy

**Fig. 5:**
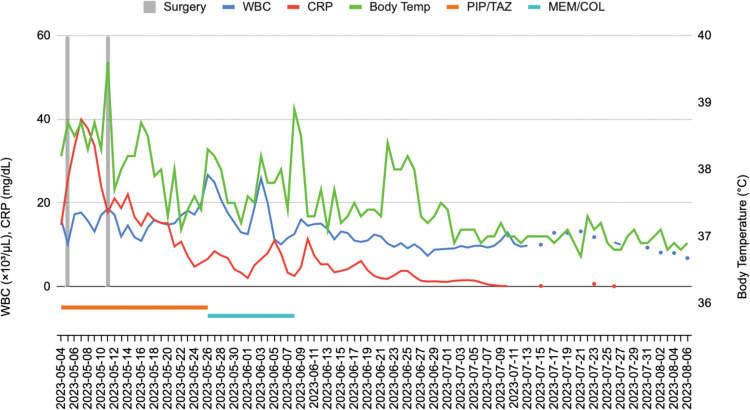
Clinical course during hospitalization. WBC count (×10³/μL) and CRP (mg/dL) values represent the measured values on each day. Body temperature is shown as the daily maximum value on the secondary y-axis. Vertical gray bars indicate the timing of surgical interventions. Horizontal bars represent the duration of antibiotic administration. Missing values indicate days on which laboratory tests were not performed. CRP, C-reactive protein; MEM/COL, meropenem plus colistin; PIP/TAZ, piperacillin–tazobactam; WBC, white blood cell

The standard approach to antibiotic therapy involves initial broad-spectrum antibiotics, followed by targeted adjustments based on culture results.^18,19)^ In this case, empirical treatment with piperacillin-tazobactam was initiated to manage generalized peritonitis. Postoperative cultures obtained from surgical drains and PCD fluid initially yielded *Escherichia coli* and *Pseudomonas aeruginosa*. Despite appropriate drainage and broad-spectrum antibiotic therapy, the patient continued to exhibit persistent fever, leukocytosis, and elevated CRP levels. Subsequent cultures from both surgical drains and percutaneous drainage fluid revealed *Acinetobacter baumannii*, prompting escalation of the antibiotic regimen to meropenem and colistin. The modified antibiotic regimen was administered for over 2 weeks, and it was discontinued based on the patient’s clinical improvement and follow-up culture results (**Fig. 5**).^20)^

### Diagnostic challenges

Early recognition and diagnosis of NF are essential due to its rapid progression and poor prognosis. The Laboratory Risk Indicator for Necrotizing Fasciitis (LRINEC) score has been proposed as a tool for predicting NF based on laboratory findings.^21)^ However, in this case, applying this criterion for diagnosis proved to be challenging. Moreover, the initial CT scan obtained upon the patient’s presentation to the emergency department did not clearly indicate NF (**Fig. 1**). Even the follow-up CT performed prior to the second surgery failed to provide definitive evidence to suspect or confirm the diagnosis (**Fig. 2**). Notably, aside from advanced age (>60), the patient had no significant comorbidities that would have placed her at a high risk for NF.^5)^ Furthermore, the shorter duration and milder severity of symptoms compared to other generalized peritonitis cases made early suspicion of NF even more difficult. Supporting this, previous studies have reported that 28.1% of patients with DNM had no underlying comorbidities, further highlighting the diagnostic difficulty in such cases.^10)^ In our patient, the clinical presentation of NF was indistinguishable from an anastomotic site leakage, adding to the diagnostic challenge.

## CONCLUSIONS

The fundamental principle of surgical debridement is the thorough removal of necrotic tissue. However, no single approach applies to all cases since the optimal strategy depends on the timing of diagnosis, the patient’s condition, the extent of infection, and the route of its spread. The extent and frequency of surgical interventions are determined based on the patient’s recovery status and the macroscopic findings of the affected tissues, relying heavily on the surgeon’s clinical judgment. While performing an extensive and thorough debridement is crucial, an overly aggressive surgical approach is not always the optimal treatment strategy.^22)^ This case report examines both ascending and descending infections in necrotizing mediastinitis. In this regard, by reviewing this case in the context of previously reported instances, we hope to provide a basis for more informed therapeutic strategies in similar situations and offer guidance for surgeons encountering comparable challenges. We acknowledge that the proposed infection pathway and supporting evidence in this report remain limited, making it difficult to derive widely applicable conclusions. However, we anticipate that this case serves as an initial step toward further research, encouraging the accumulation of additional cases and the development of a stronger evidence base.

## DECLARATIONS

### Availability of data and materials

All data supporting the conclusions of this article are included within the manuscript.

## References

[1] Stevens DL, Bryant AE. Necrotizing soft-tissue infections. N Engl J Med 2017; 377: 2253–65. 29211672 10.1056/NEJMra1600673

[2] Das DK, Baker MG, Venugopal K. Increasing incidence of necrotizing fasciitis in New Zealand: a nationwide study over the period 1990 to 2006. J Infect 2011; 63: 429–33. 21864570 10.1016/j.jinf.2011.07.019

[3] Cocanour CS, Chang P, Huston JM, et al. Management and novel adjuncts of necrotizing soft tissue infections. Surg Infect (Larchmt) 2017; 18: 250–72. 28375805 10.1089/sur.2016.200 PMC5393412

[4] Mills MK, Faraklas I, Davis C, et al. Outcomes from treatment of necrotizing soft-tissue infections: results from the National Surgical Quality Improvement Program database. Am J Surg 2010; 200: 790–6; discussion 796–7. 21146022 10.1016/j.amjsurg.2010.06.008

[5] Falconi S, Wilhelm C, Loewen J, et al. Necrotizing fasciitis of the abdominal wall secondary to complicated appendicitis: a case report. Cureus 2023; 15: e39635. 37388614 10.7759/cureus.39635 PMC10305508

[6] Lin MS, Chu YC, Ho MP. Abdominal-wall necrotizing fasciitis in an elderly woman. Asian J Surg 2024; 47: 515–6. 37813793 10.1016/j.asjsur.2023.09.164

[7] Santarrufina-Martínez S, Lissón MMZY, Primo-Romaguera V, et al. Necrotizing fasciitis of the thigh secondary to perforated rectal cancer: the sciatic foramen as a route for infective spread. Cir Cir 2024; 92: 264–6. 38782396 10.24875/CIRU.21000843

[8] Piedra T, Martín-Cuesta L, Arnáiz J, et al. Necrotizing fasciitis secondary to diverticulitis. Emerg Radiol 2007; 13: 345–8. 17216174 10.1007/s10140-006-0566-9

[9] Pantelić M, Stojić MS, Petrović Đ, et al. Necrotizing fasciitis after total abdominal hysterectomy: a case report. Medicine (Baltimore) 2023; 102: e34451. 37543829 10.1097/MD.0000000000034451 PMC10403000

[10] Sumi Y. Descending necrotizing mediastinitis: 5 years of published data in Japan. Acute Med Surg 2014; 2: 1–12. 29123684 10.1002/ams2.56 PMC5667189

[11] Dajer-Fadel WL, Pichardo-González M, Estrada-Ramos S, et al. Ascending necrotizing mediastinitis secondary to emphysematous pyelonephritis. Asian Cardiovasc Thorac Ann 2014; 22: 869–71. 24887856 10.1177/0218492313495860

[12] Sánchez-Matás C, Aldabó-Pallas T, Palacios-García I, et al. Ascending necrotizing mediastinitis: an exceptional case. Arch Bronconeumol 2021; 57: 780–2. 35698993 10.1016/j.arbr.2021.10.007

[13] Chang YC, Chen CW. Thoracoscopic drainage of ascending mediastinitis arising from pancreatic pseudocyst. Interact Cardiovasc Thorac Surg 2009; 9: 144–5. 19386659 10.1510/icvts.2009.202226

[14] Chong BK, Yun JK, Kim JB, et al. Multiple ascending aortic mural thrombi and acute necrotizing mediastinitis secondary to acute pancreatitis. Korean J Thorac Cardiovasc Surg 2016; 49: 401–4. 27734004 10.5090/kjtcs.2016.49.5.401 PMC5059130

[15] Watanabe M, Ohshika Y, Aoki T, et al. Empyema and mediastinitis complicating retropharyngeal abscess. Thorax 1994; 49: 1179–80. 7831642 10.1136/thx.49.11.1179 PMC475289

[16] Davoodabadi A, Entezari H, Talari H, et al. Acute purulent mediastinitis with sequential bilateral pleural empyema caused by neck trauma: a unique occurrence, a case report. Int J Surg Case Rep 2019; 65: 171–5. 31715448 10.1016/j.ijscr.2019.10.065 PMC6849119

[17] Dalbem CS, Nunes TF, Machado MS, et al. Pneumomediastinum and pneumoretroperitoneum: an extremely rare presentation of acute appendicitis. BMJ Case Rep 2015; 2015: bcr2014207255. 10.1136/bcr-2014-207255 PMC4289810 25576508

[18] Anaya DA, Dellinger EP. Necrotizing soft-tissue infection: diagnosis and management. Clin Infect Dis 2007; 44: 705–10. 17278065 10.1086/511638

[19] Salati SA. Necrotizing fasciitis: a review. Pol Przegl Chir 2022; 95: 1–8. 10.5604/01.3001.0015.7676 36805313

[20] Lauerman MH, Kolesnik O, Sethuraman K, et al. Less is more? Antibiotic duration and outcomes in Fournier’s gangrene. J Trauma Acute Care Surg 2017; 83: 443–8. 28538648 10.1097/TA.0000000000001562

[21] Wong CH, Khin LW, Heng KS, et al. The LRINEC (Laboratory Risk Indicator for Necrotizing Fasciitis) score: a tool for distinguishing necrotizing fasciitis from other soft tissue infections. Crit Care Med 2004; 32: 1535–41. 15241098 10.1097/01.ccm.0000129486.35458.7d

[22] Sun X, Xie T. Management of necrotizing fasciitis and its surgical aspects. Int J Low Extrem Wounds 2015; 14: 328–34. 26597210 10.1177/1534734615606522

